# Supplementary material for: Splice-Junction-Based Mapping of Alternative Isoforms in the Human Proteome
Source: Cell Rep. Author manuscript; Available in PMC 2020 Jan 15. (PMC6961840; doi:10.1016/j.celrep.2019.11.026)
Supplement: 2 [file NIHMS1546469-supplement-2.zip › DF1/PXD010154/Heart/Heart_89_STRN4_HPVPAGFSFVAAAASSCRPLGSGAGPGPTGAAPVSAPAPGPGPAGKGGGGGGSPG.pdf]

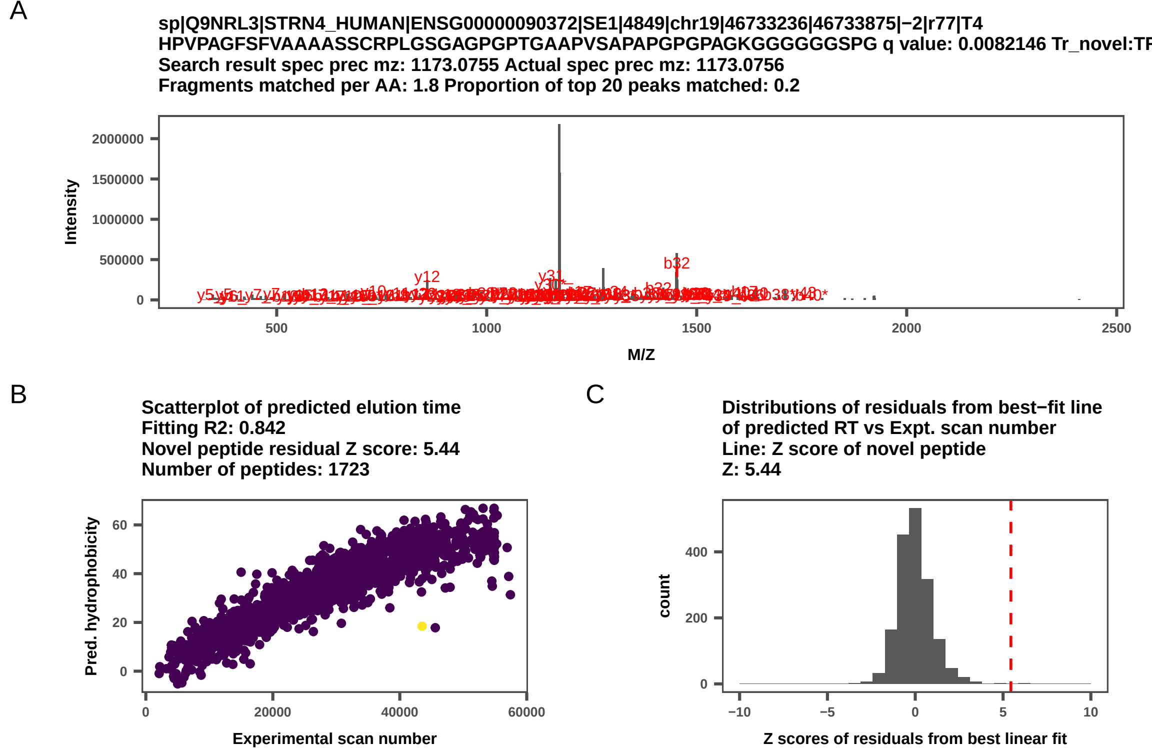
A
sp|Q9NRL3|STRN4_HUMAN|ENSG00000090372|SE1|4849|chr19|46733236|46733875|−2|r77|T4
HPVPAGFSFVAAAASSCRPLGSGAGPGPTGAAPVSAPAPGPGPAGKGGGGGGSPG q value: 0.0082146 Tr_novel:TF
Search result spec prec mz: 1173.0755 Actual spec prec mz: 1173.0756
Fragments matched per AA: 1.8 Proportion of top 20 peaks matched: 0.2
Intensity
2000000
1500000
1000000
500000
0
b32
y12
M/Z
500
1000
1500
2000
2500
B
Scatterplot of predicted elution time
Fitting R2: 0.842
Novel peptide residual Z score: 5.44
Number of peptides: 1723
Pred. hydrophobicity
60
40
20
0
Experimental scan number
0
20000
40000
60000
C
Distributions of residuals from best−fit line
of predicted RT vs Expt. scan number
Line: Z score of novel peptide
Z: 5.44
count
400
200
0
Z scores of residuals from best linear fit
−10
−5
0
5
10
